# Roles of mediodorsal thalamus in observational fear-related neural activity in mouse anterior cingulate cortex

**DOI:** 10.1186/s13041-025-01188-9

**Published:** 2025-02-25

**Authors:** Kritika Ramesh, Indrajith R. Nair, Naoki Yamamoto, Sachie K. Ogawa, Joseph I. Terranova, Takashi Kitamura

**Affiliations:** 1 Department of Psychiatry, University of Texas Southwestern Medical Center, Dallas, TX USA; 2 Department of Biology, Kyushu University, Fukuoka, JPN Japan; 3 Peter O’Donnell Jr. Brain Institute, University of Texas Southwestern Medical Center, Dallas, TX USA; 4 Department of Anatomy, Midwestern University, Downers Grove, IL USA; 5 Department of Neuroscience, University of Texas Southwestern Medical Center, Dallas, TX USA

**Keywords:** Anterior cingulate cortex, Mediodorsal thalamus, Observational fear, Empathy, Calcium imaging, Optogenetic inhibition

## Abstract

Observational fear (OF) is the ability to vicariously experience and learn from another’s fearful situation, enabling adaptive responses crucial for survival. It has been shown that the anterior cingulate cortex (ACC) and basolateral amygdala (BLA) are crucial for OF. A subset of neurons in the ACC is activated when observing aversive events in the demonstrator, which elicits OF. However, the neural circuit mechanisms underlying the expression of OF-related activity in the ACC remain unexplored. Previous studies have shown that the mediodorsal thalamus (MD) is crucial for OF, and MD neurons project to the ACC. Therefore, we hypothesize that the projection from MD to ACC may facilitate the OF-related activity in the ACC. By utilizing *in vivo* calcium imaging combined with the optogenetic terminal inhibition of MD-ACC pathway, we found that a subset of ACC neurons was activated when observing demonstrator’s fearful situation in male mice. Furthermore, the optogenetic inhibition of the MD-ACC projection during the demonstrator’s aversive moments significantly suppressed the OF-related activity in the ACC. Our data suggests that the MD-ACC projection plays a role in OF-related activity in ACC neurons.

**Supplementary Information:**

The online version contains supplementary material available at 10.1186/s13041-025-01188-9.

## Introduction

Fear is essential for the survival of individuals and groups, as it alerts us to possible danger in our current situations [1, 2]. However, we often experience fear even when we are not directly involved in a dangerous situation – rather, we observe another individual showing fear and, in turn, we experience fear as well [3]. This phenomenon is known as observational fear (OF), which is considered as an empathic response where the expression of fear by a conspecific in an aversive situation induces a fear response in another individual [4–6]. OF has been previously identified in rodents, in that individuals witnessing a distressed conspecific showed higher fear and anxiety related behaviors, such as freezing or crouching [7, 8], even without direct interaction with the aversive stimulus [9, 10].

It has been demonstrated that the anterior cingulate cortex (ACC), the basolateral amygdala (BLA) and the projection from ACC to BLA are critical for OF [5, 9, 11–22]. Electrophysiological studies showed that a subset of neurons in the ACC is activated when an individual observes an aversive shock delivered to the demonstrator [11, 23]. Furthermore, the optogenetic inhibition of the ACC-BLA pathway during the demonstrator’s aversive moments reduced OF [19]. These results suggest that OF-related activity in the ACC may be crucial for OF. However, the neural circuit mechanisms underlying the expression of OF-related neural activity in the ACC remain unknown.

Along with the ACC and BLA, the mediodorsal thalamus (MD) has been shown to play a critical role in OF [9, 13, 24]. The MD is known to project to the ACC across different species [13, 25–28]. Furthermore, previous studies identified that pain signals are transmitted via lateral and medial pain systems with the thalamus being an essential relay point between the spinal cord and cortex [29]. Specifically, nociceptive stimuli, or pain stimuli, are transmitted to the prefrontal cortex from the midline and intralaminar thalamic nuclei [30] that lie adjacent to the MD, which is one of the primary points of projection to the ACC [9]. Therefore, we hypothesize that the projection from MD to ACC may facilitate the OF-related activity in the ACC, which would trigger the OF response. To test this, we employed the previously established OF model in mice [19] to investigate how inhibiting MD projections to the ACC during OF affects neural activity in the ACC as well as OF. We recorded neural activity in the ACC using *in vivo* calcium imaging during OF and examined the effects of optogenetic inhibition of MD-ACC pathway during the demonstrator’s aversive moments on the OF-related ACC activity as well as OF response.

## Results

### Projection from MD to ACC in mice

**Fig. 1:**
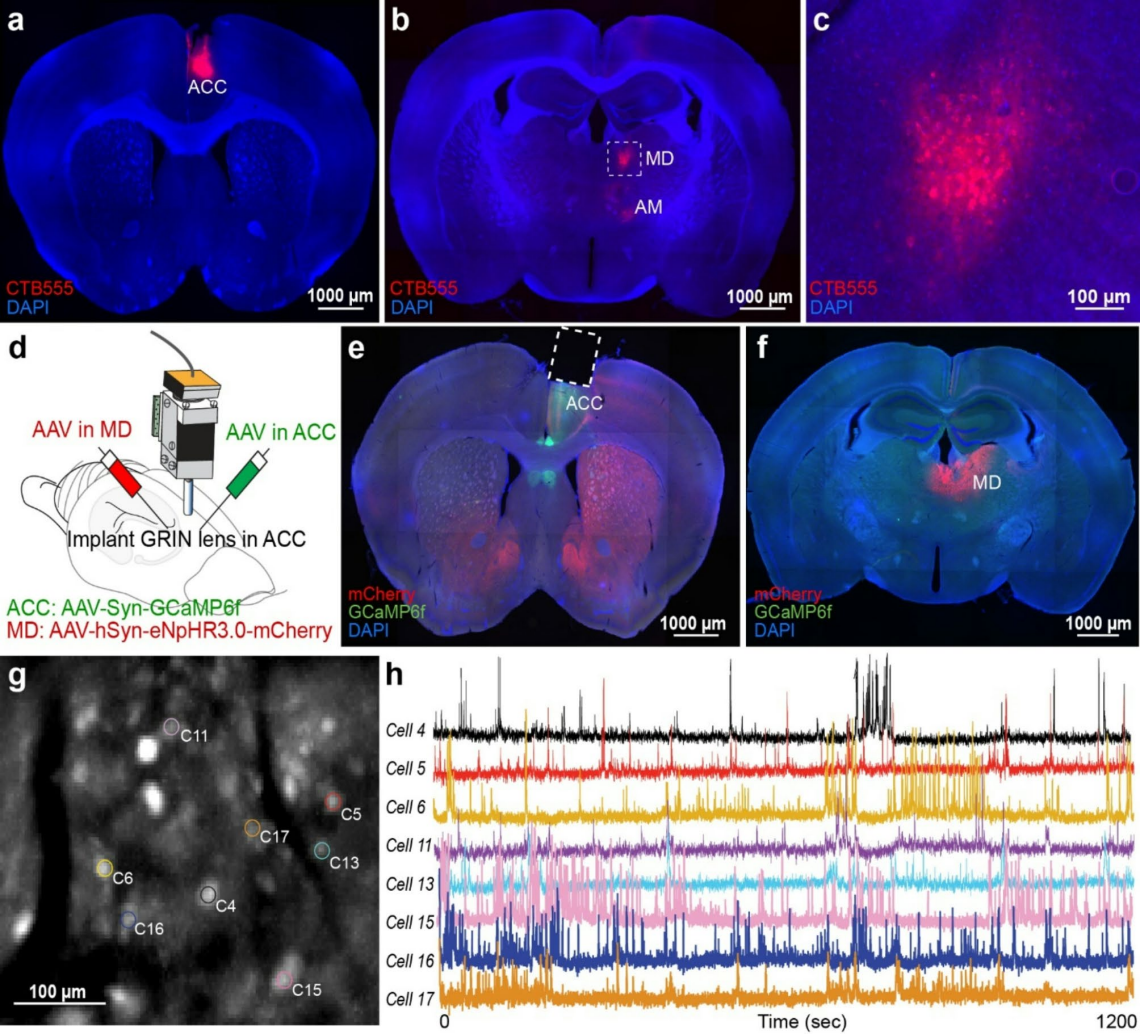
Retrograde tracing of MD-ACC pathway and *in vivo* calcium imaging in ACC. (**a**) Coronal section of ACC. CTB-555 (red) injection into ACC. DAPI (blue). (**b**) Coronal section of MD. CTB-555 (red). DAPI (blue). (**c**) Magnified image of MD from (**b**). (**d**) Viral injections (AAV_2/5_-Syn-GCaMP6f into ACC, AAV_2/5_-hSyn-eNpHR3.0-mCherry into MD) and GRIN lens implantation into ACC. (**e**) Viral expression and lens placement site at ACC. Coronal section of GRIN lens implanted animal. DAPI (blue). GCaMP6f (green). eNpHR3.0-mCherry (red). Dotted rectangle represents the position of GRIN lens. (**f**) AAV injection site at MD. (**g**) Maximum projection image of GCaMP6f-expressing cells acquired through a micro-GRIN-lens over 20 min from the ACC during the OF protocol. (**h**) Sample calcium traces in 8 cells. Calcium transients correspond to neural activity of the cells

To demonstrate the projection from MD to ACC in mice, we injected CTB-555, a retrograde tracer, into the ACC (Fig. 1a). We found CTB-positive cells in MD, indicating that the MD neurons project to the ACC in mice (Fig. 1b-c). We also found sparsely CTB-positive cells in anteromedial thalamus (AM), indicating that AM neurons also project to the ACC, which is consistent with previous reports [13, 31] (Fig. 1b).

### ACC neural activity during OF

**Fig. 2:**
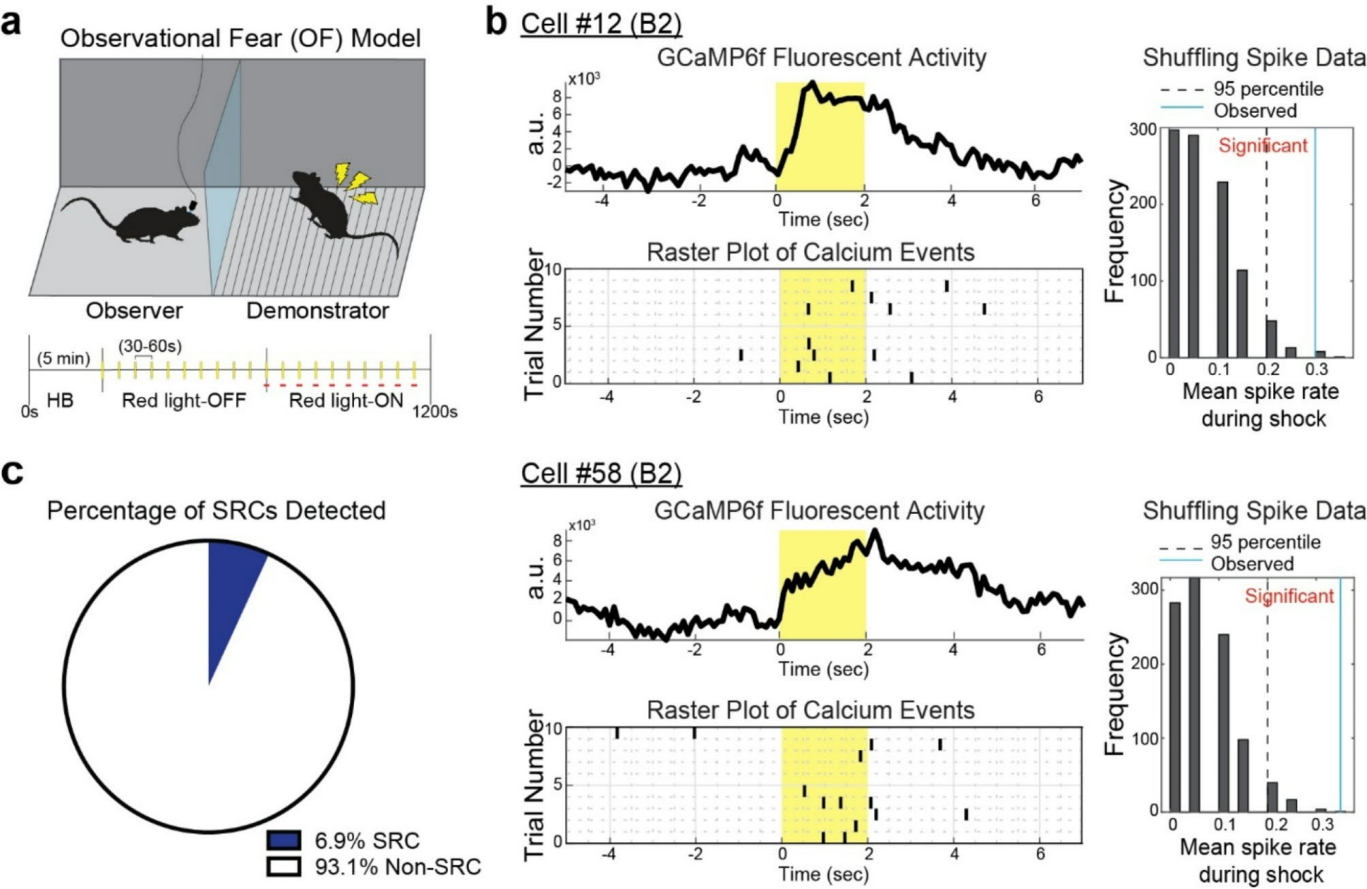
Shock Responding Cell (SRC) in mouse ACC. (**a**) OF behavioral model and experimental schedule. The observer mouse is placed across a stranger demonstrator who is exposed to the shock grid. After a 5 min habituation (HB) period, the demonstrator receives 20 electrical foot-shocks separated by 30–60 s each. Red LED is activated during the second 10 shocks (light-ON condition) to inhibit MD-ACC pathway. (**b**) Representative data from two detected SRCs. Left top panel) Mean of GCaMP6f fluorescent activity traces over 10 red light-OFF trials. Yellow color bar indicates the shock period (2 s). Left bottom panel) Raster plots of calcium transients before and after the shock moment over 10 shocks during red light-OFF condition. Black line plots indicate the event timing of calcium transients. Yellow color bar indicates the shock period (2 s). Right panels) Frequency distribution of mean spike rate determined by 1000 times shuffle. Dashed line indicates 95th percentile of the frequency distribution. Blue line indicates mean spike rate observed during shock moment. (**c**) 27 cells (6.9%) of the total 391 cells detected over 3 mice were identified as SRCs

To identify the OF-related activity in the mouse ACC, we examined *in vivo* calcium imaging from the right hemisphere of ACC in the observer during OF by expressing GCaMP6f, a calcium indicator, in the ACC and implanting a GRIN-lens into the ACC (Figs. 1d-h and 2a). We detected a total of 391 GCaMP-expressing cells from 3 mice over total 5 sessions in the ACC during OF protocol through a head-mounted miniatured fluorescent microscopy (Fig. 1g-h). Previous electrophysiological studies have found that ACC neurons show heightened activity during the shock delivery moment in mice and rats, making them shock-responding cells (SRCs) [11, 23]. Consistent with the previous reports, we observed a subset of ACC neurons that increased calcium activity from baseline during the shock moment to the demonstrator (Fig. 2b, Supplemental Fig. 1). To quantitatively identify SRCs, we measured the number of calcium transients per cell during OF and examined if the calcium transients in each cell significantly occurs during shock moment, determined by shuffling analysis (Fig. 2b). We found that 27 out of the 391 detected neurons (6.9%) were determined to be SRCs (Fig. 2c). These results indicate a subset of ACC neurons shows the OF-related activity in mice. We also analyzed shock-suppressed cells (SSCs) which was previously reported in the BLA during experience-dependent OF protocol [32]. In contrast to BLA, we did not observe shock-suppressed cells.

### Effects of optogenetic terminal inhibition of MD-ACC pathway on OF-related neuronal activity in the ACC

**Fig. 3:**
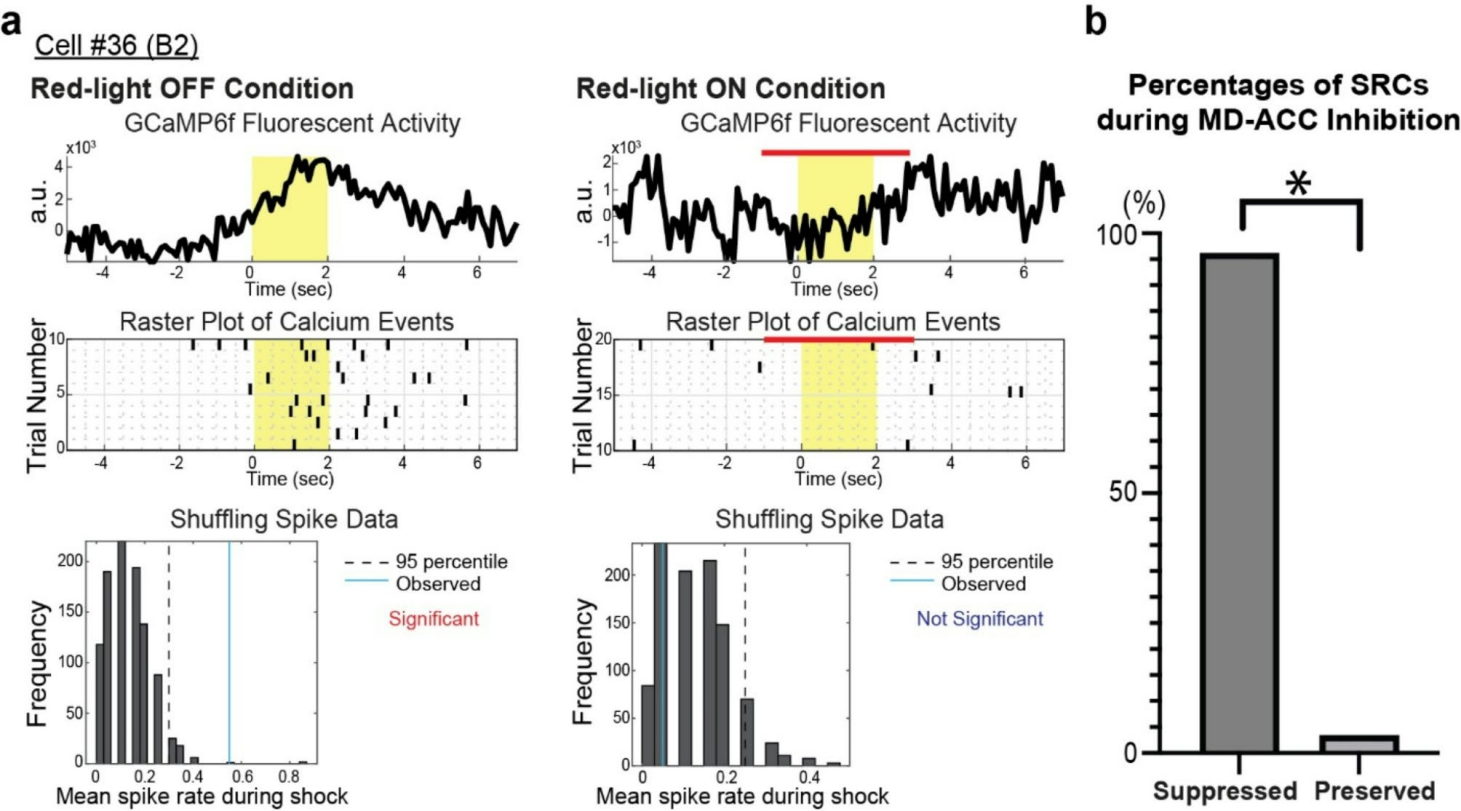
Effects of optogenetic inhibition of MD-ACC pathway on shock-responding activity in observer’s ACC. (**a**) Representative data from Cell #36(B2). Top panel) Mean of GCaMP6f fluorescent activity traces over light-OFF condition (left) and light-ON (right) condition. Yellow color bar indicates the shock period (2 second). Red color bars indicate the red-LED ON period. Middle panel) Raster plots of calcium transients before and after the shock moment over 10 shocks during red light-OFF (left) and light-ON (right) condition. Black line plots indicate the event timing of calcium transients. Yellow color bar indicates the shock period (2 second). Red color bars indicate the red-LED ON period. Bottom panels) Frequency distribution of mean spike rate determined by 1000 times shuffle. Dashed line indicates 95th percentile of the frequency distribution. Blue line indicates mean spike rate observed during shock moment during red light-OFF and light-ON condition. (**b**) Percentages of 27 SRCs from 3 mice (Fig. 2c) during MD terminal inhibition in ACC (96.4%, *p* < 0.0001, X^2^ = 23.148, df = 1)

Next, we examined the role of the MD-ACC pathway on the shock-responding activity in ACC neurons by using eNpHR3.0-mCherry expressed in the right hemisphere of MD neurons (Fig. 1f). We optogenetically inhibited the MD-ACC pathway during the demonstrator’s shock moments by illuminating red LED into the right hemisphere of ACC (red light-ON condition) and examined the corresponding effect on the activity of SRCs identified during the red light-OFF condition to determine if their shock-responding activity was altered during the inhibition. We found that 26 out of 27 SRCs (96.4%) showed suppression of the shock responding activity during red light-ON condition (Fig. 3a-b). These cells also showed either decreased or baseline GCaMP6f activity during the inhibition of MD-ACC pathway (Fig. 3a). These results suggest that MD-ACC pathway is crucial for shock-responding activity in the ACC. Of note, we also observed a secondary population of SRCs: these cells showed no significant increase in activity during the shock moment in the OFF condition, but had significantly increased activity in the ON condition (Supplemental Fig. 2).

### Effects of optogenetic terminal inhibition of MD-ACC pathway on OF

**Fig. 4:**
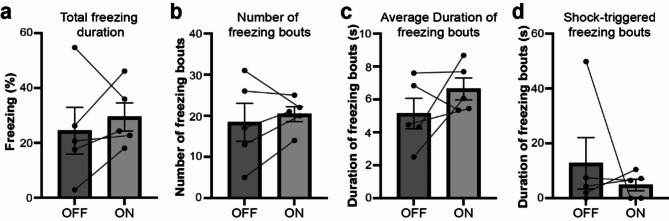
Effects of optogenetic inhibition for MD-ACC pathway on OF. (**a**) Averaged percentages of observer freezing behavior during red light-OFF and red light-ON condition (t = 0.7464, df = 4, *P* = 0.4969). (**b**) Averaged number of observer freezing bouts during OFF and ON condition (t = 0.6202, df = 4, *P* = 0.5687). (**c**) Averaged duration of observer freezing bouts during OFF and ON condition (t = 1.377, df = 4, *P* = 0.2407). (**d**) Averaged freezing duration of freezing bouts beginning in the shock moment during OFF and ON condition (t = 0.7468, df = 4, *P* = 0.4967)

A previous study showed that inhibition of the MD with lidocaine impairs OF behavior [9]. We examined the total duration of freezing response in the observer during red light-OFF condition and light-ON condition. We did not see significant differences in the average freezing duration between red light-OFF condition and light-ON condition time periods (Fig. 4a). We also did not observe a difference in the number and duration of freezing epochs during red light-OFF condition and light-ON condition (Fig. 4b-c). Freezing epochs were defined as bouts of freezing that began during the designated time period. We then analyzed the duration of freezing epochs beginning during the shock period and did not find a significant difference between red light-ON and light-OFF conditions (Fig. 4d).

## Discussion

In this study, we found that a subpopulation of ACC neurons exhibit shock-responding activity during OF in mice (Fig. 2). The shock-responding activity of these cells is significantly suppressed during the optogenetic inhibition of MD-ACC pathway, suggesting that the MD-ACC pathway mediates shock-responding activity in the ACC during OF (Fig. 3). While previous studies have established that the ACC neurons show the shock-responding activity [11, 23] and that the MD inhibition with lidocaine [9] or chemical lesion with ibotenic acid [13] impairs OF, our findings link these studies and reveal that the MD projection to ACC is the crucial pathway for the OF-related neural activity in the ACC.

Both rats and mice show robust freezing by observing other’s fearful situation [33]. However, the amplitude of the freezing response in the observer mouse versus rat is different; the observer rats exhibit a higher freezing response than observer mice [33]. In this study, we found that the subpopulation of neurons in the ACC that were identified as shock responding cell in mice was much smaller (6.9%) compared with the previous study in rats (61.7%) [23]. Our findings of these smaller number of SRCs in the mouse ACC may be related to the difference in robustness of the OF response.

While we found that the optogenetic inhibition of MD-ACC pathway during the shock moment significantly suppressed the shock responding activity in the ACC (Fig. 3), what information do MD neurons send to ACC to trigger OF response? Since previous studies have identified OF response is visual stimuli-dependent, as replacing the clear partition between chambers with an opaque partition significantly decreases the OF response in the observer [9, 34], we assume that the visual input of the demonstrator’s exhibition of fear triggers SRC activity in the observer’s ACC. It has been shown previously that given the current experimental setup, where both observer and demonstrator can move freely within their chambers, the observer mouse visually attends to the demonstrator during demonstrator shock moments [19]. Therefore, MD may send the OF-related visual stimulus to ACC. Furthermore, the thalamus is known to be an intermediate between the spinal cord and cortex, especially for pain stimuli that are transmitted to the cortex via thalamic nuclei adjacent to the MD [9, 29, 30] Therefore, MD may convey visually triggered-pain perception information to ACC, which would elicit OF-related activity in ACC and OF behavior. Further studies will be required for the mechanistic understanding about how ACC neurons express OF-related activity.

In this study, we did not observe the effects on OF behavior (Fig. 4). Since previous studies showed right but not left ACC is crucial for OF response [13], the unilateral red-light illumination of ACC should be effective for OF behavior and therefore we assume that the failure of the effects might be due to the partial illumination of the red light into the ACC through a GRIN lens, as the ACC is a large structure extending along the anterior-posterior axis. Given that the ACC receives projections from multiple thalamic subregions [31], it is also possible that alternate inputs to the ACC or projections from the MD to other regions [13] may be compensating for the MD-ACC inhibition, therefore maintaining the behavioral response. However, considering that both MD and ACC are crucial for the OF response [9], it is likely that more complete inhibition of MD projections in the ACC would decrease OF behavior. Further study would be necessary to investigate the roles of MD-ACC pathway on OF response.

We found an interesting pattern of ACC activity that shows an increase in neuron activity during MD inhibition in some cells compared with the red light-OFF condition (Supplemental Fig. 2). This result may point towards the potential involvement of an inhibitory pathway between the MD and the ACC. Previous studies have shown that there is a direct inhibitory link between the MD and ACC via inhibitory interneurons that drive this feed-forward inhibition [27, 35], and as such, it is possible that these inhibitory neurons may play a role in modulating ACC activity during OF, and that their activity is further controlled by the MD under normal conditions. Upon inhibition of the MD, these interneurons may no longer be activated, resulting in the increased ACC activity during shock moment that we observed. Further studies will be required to identify the potential role of these interneurons on both neural activity in the ACC and on OF behavior in the observer.

## Materials and methods

### Animals

All experiments were performed with C57BL/6J male mice. All mice were between 9 and 20 weeks old at the time of experimentation. Prior to microinjection and surgical implantation of calcium imaging equipment, observer mice were housed with between 2 and 5 littermates. Following microinjection and implantation, mice were single-housed to prevent alteration of implant by littermates. Demonstrator mice were also group-housed prior to experimentation, then single-housed following the first experimental trial. A 12-hour light/dark cycle was automatically maintained in the housing facility. Food and water were available *ad libitum.* Experiments were conducted during light cycle. All protocols regarding mouse use and treatment aligned with NIH guidelines, and procedures were approved by UT Southwestern Institutional Animal Care and Use Committee (IACUC).

### Observational fear protocol

The observational fear (OF) apparatus is a fear conditioning box divided into two chambers by a transparent plexiglass partition (observer chamber: 15 cm W x 20 cm D x 20 cm H; demonstrator chamber: 15 cm W x 20 cm D x 20 cm H), as we previously demonstrated [19, 20]. In the observer chamber, there was an opaque plexiglass floor to prevent the observer from seeing the shock grid or inadvertently experiencing shock. In the demonstrator chamber, the stainless-steel rod floor was exposed, which delivered mild shocks to the demonstrator. The OF apparatus was housed inside a soundproof fear conditioning chamber (Med Associates) to prevent external noise. The naive experimental mouse was placed in the observer chamber, while the stranger conspecific was placed in the Demonstrator chamber. Both mice underwent a 5-minute habituation period, in which they were allowed to freely explore their respective chamber. Following the habituation period, there is a 15-minute demonstrator shock period in which the demonstrator mouse received a series of 20, 1.0 mA 2-second foot shocks, with a 30–60 s interval between each shock (Fig. 2a). The interval time was randomized between each shock moment to prevent the demonstrator mouse from anticipating the shock. The protocol was repeated for a total of 3 sessions per mouse, with a minimum of 4 h between each session. A maximum of 2 sessions were conducted in a single day to prevent photobleaching of cells. The same demonstrator mouse was used for all 3 sessions of a single observer mouse. The shock protocol was designed and controlled digitally on the VideoFreeze Video Fear Conditioning Software (Med Associates). All behavioral chambers were cleaned between trials with PV-15.

### General stereotaxic surgery protocols

All surgeries were performed using a digital small animal stereotax (David Kopf Instruments) with a microscope (Leica) attached. Prior to surgery, mice were anesthetized with isoflurane (4% for induction, 1–2.5% for maintenance). Microinjections were performed using 10uL microsyringe (Hamilton) flush with mineral oil and a glass micropipette (Drummond Scientific Company), and injection volume/speed was controlled digitally via microsyringe pump (World Precision Instruments). The micropipette remained in the tissue for 5 min post-injection before removing the injection needle from the tissue. Following surgery, mice were given the analgesics buprenorphine SR and meloxicam (2 mg/kg) and remained on a heating pad to rest until fully recovered from the anesthesia. All surgery procedures were approved by UT Southwestern IACUC, and followed NIH guidelines on aseptic technique.

### Stereotaxic surgery for CTB injection, optogenetic inhibition and *in vivo* calcium imaging

To visualize the ACC input neurons, we injected CTB-555 (200 nL/injection, 3.0 nL/sec) into the right ACC at the following coordinates relative to bregma: AP: +1.0 mm, ML: + 0.3 mm, DV: -1.5 mm, as we previously demonstrated [36, 37]. Following a 1 week incubation period, the tissue was perfused and counterstained with DAPI (Fig. 1a).

To express GCaMP6f, a calcium indicator, in the ACC, we injected AAV_2/5_-Syn-GCaMP6f (500 nL/injection, 3.0 nL/sec, 1.3 × 10^13^ GC/mL) to the right ACC at the following coordinates relative to bregma: AP: +1.0 mm, ML: + 0.3 mm, DV: -1.5 mm (Fig. 1d-e). To inhibit the MD-ACC pathway, we also injected AAV_2/5_-hSyn-eNpHR3.0-mCherry (200 nL/injection, 3.0 nL/sec, 4.4 × 10^12^ mol/mL) into the right MD at the following coordinates relative to bregma: AP: -1.4 mm, ML: +0.3 mm, DV: -3.5 mm (Fig. 1d and f). After 4 weeks, we implanted a GRIN lens (1 mm diameter, NA: 0.5) to the ACC at the following coordinates relative to bregma: AP: +1.0 mm, ML: +0.5 mm, DV: − 1.0 mm (Fig. 1d-e). Lenses were implanted with an angle of 4 degrees to avoid damaging the superior sagittal sinus. 8 screws on the skull and dental cement (C&B Metabond, Parkell) were used to secure the lens. 2 weeks following lens implant, an Inscopix baseplate was attached over the lens using UV activated glue (Norland Optical) to serve as a connection point between the imaging camera and the lens.

### Calcium imaging and optogenetic inhibition during OF protocol

During the OF behavioral protocol, calcium activity in the ACC neurons was captured using a miniature fluorescent microscope (Inscopix nVoke2) as we previously demonstrated [19, 38–41]. Throughout the entire 20-minute protocol, the imaging camera is connected to a lens implanted in the ACC and illuminates the region via blue LED. During each individual shock moment (2 s shock delivery to the demonstrator), a red LED is also shined on the ACC via the imaging camera to stimulate the halorhodopsin for the terminal inhibition of MD neurons projecting to the ACC. The red LED (620 ± 30 nm excitation and 1.0 mW) is turned on one second before the shock moment and is sustained through one second after the shock moment (total 4 s) to account for any anticipation or delay in MD activity. The red LED was off for the first 10 shock moments and on for the second 10 shock moments. All calcium imaging recordings were obtained at 20 Hz and 0.3 mW (LED power). The calcium imaging recording was initiated prior to starting the experimental protocol on the VideoFreeze software. The exact timestamp of the start of the experiment was recorded via a general-purpose input/output (GPIO) input trigger.

### Calcium imaging analyses

Of the total 9 recorded experimental sessions from 3 mice, 5 sessions were used for analysis. The remaining 4 session recordings were not used due to issues in recording, such as shifting of the camera during recording or changes in the focus of FOV during the experiment. Cells obtained from each session were considered as individual sets. After obtaining the raw calcium imaging recordings from each session, we processed them using the Inscopix Data Processing Software (IDPS). The initial processing steps included preprocessing of the data, spatial bandpass, and motion correction to eliminate any noise/external influences on the data. Data was not down sampled during processing. Following motion correction, recordings were processed via a maximum intensity projection, which was used for the PCA-ICA analysis on the recording with the maximum number of iterations limited to the approximate total number of cells from the maximum intensity projection. This single cell identification method first identifies each “cell” by isolating regions of interest (ROI) in each frame of the recording that show significant fluctuations in fluorescence and meet the cell identification parameters (Cell Size: 7 pixels < x < 49 pixels). Parameters for fluorescence changes for PCA-ICA were as follows: ICA random seed = 0, ICA convergence threshold = 0.00001, Block Size = 1000, ICA unmitigated dimensions = spatial, ICA temporal weights = 0.00. After identifying ROIs, the individual calcium transients for each ROI is obtained, which corresponds to the changes in fluorescence over the experimental time period. Each cell and calcium transient was manually checked to confirm that the identified ROI was truly a cell, rather than light artifact or noise. The accepted cell traces were exported for further analysis using our custom-made MATLAB scripts.

In MATLAB, our script first takes the timestamp for the start of the experiment from the associated GPIO file which records the start and end of the experimental time frame. Using these values, the cell trace recordings are aligned to the exact time period of the experiment to avoid any errors in syncing cellular activity to experimental schedules. Then, the program iterates through each identified cell’s trace and calculates the timestamps at which calcium “spikes” occurred. Calcium spikes were defined as events where the peak fluorescence exceeded a threshold of 2 standard deviations above the baseline mean. These spikes are indicative of cellular activity, as the increase in fluorescence is due to a large volume of Ca^2+^ entering the neuron.

Once the significant spikes are identified, the exact timestamps of these spikes are determined from the original cell trace values and catalogued. Following this, the shuffling or randomization method of significance was used to determine if calcium spikes that were determined to be “responding” to the shock moments were corresponding with the OF response or by chance. In the shuffling method, the mean spike rate of each neuron was calculated during the shock period. Then, the spiking pattern was “shuffled”: all spikes were shifted temporally by a random time increment between 20 and 1200 s. The mean spike rate during shock moment in red light-OFF condition was then recalculated. This procedure was conducted 1000 times, creating a distribution of mean spike rates. The observed spike rate was compared to the 95th percentile of the shuffled spike rate distribution to determine the significance of spike activity, with significance indicating response to the demonstrator’s shock rather than by chance. Once the shock responding cells were identified in the red light-OFF condition (Fig. 2), a similar spike fluorescence analysis and shuffling analysis was performed on these cells during the red light-ON condition to assess for suppression (Fig. 3). A cell was suppressed by MD terminal inhibition in ACC if the change in spiking activity during the shock moment was not significant from baseline, and the spiking activity was no longer correlated to the shock moment. For assessing suppression for Shock Suppressed Cells (SSCs), the observed spike rate in the red-light OFF condition was compared to the 5th percentile of the shuffled spike rate distribution. Cells were considered to be suppressed during the shock moment during red light-OFF if spike rate was less than the 5th percentile of the shuffled spike rate distribution, indicating suppression of activity was in response to demonstrator’s shock.

### Behavior analyses

The behavior of both the observer and demonstrator was recorded throughout the duration of each experiment via the VideoFreeze Video Fear Conditioning Software (Med Associates). Using the BORIS (Behavioral Observation Research Interactive Software) event-logging platform, movement, freezing, and jumping epochs for both the observer and demonstrator were manually scored for each of the 5 accepted sessions, as we previously demonstrated [19, 20]. Freezing bouts were defined as when the animal exhibited no movement except for respiration. After scoring, freezing periods were further classified as bouts with duration greater than 2 s. Total freezing duration was quantified by calculating the sum of all accepted freezing bouts during a given time frame. Shock-responding freezing duration considered only those bouts which began within the 2 s shock window. Behavior analysis was not performed on recording sessions that were not used for calcium imaging analysis.

### Statistics

All graphical data is presented as mean values with Standard Error of the Mean (SEM) displayed. Graphpad Prism 10 software was used for all statistical analysis. The shuffling method was used to determine significance of correlation between spiking activity and the shock period. Two-sided chi-square test was used to determine significance of the number of SRCs affected by the optogenetic inhibition. Two-tailed paired t-test was used to determine the significance of the differences in total freezing duration, number of freezing bouts, average freezing bout duration, and average duration of shock period freezing bouts. The null hypothesis was rejected at *P* < 0.05 for all statistical analysis. Spike detection and analysis were performed in MATLAB version R2023a.

## Electronic Supplementary Material

Below is the link to the electronic supplementary material.

**Supplementary Material 1**: **Supplemental fig. 1**: (related to Fig. 2). Trial by trial visualization of calcium activity and calcium transients in SRCs and Non-SRCs. a) Representative data of SRCs from Cell #12 (B2) and Cell #58 (B2). Top panel: mean calcium transient over all 10 demonstrator shock moments in red light-OFF condition. Bottom panels (Trial 1–10): calcium transients during each demonstrator shock period. Significant spikes marked with blue stem plot. Yellow color bar indicates the shock period (2 s). b) Representative data of Non-SRCs from Cell #13 (B2) and Cell #29 (B2). Top panel: mean calcium transient over all 10 demonstrator shock moments in red light-OFF condition. Bottom panels (Trial 1–10): calcium transients during each demonstrator shock period. Significant spikes marked with blue stem plot. Yellow color bar indicates the shock period (2 s). **Supplemental fig. 2**: (related to Fig. 3). Activation of ACC SRCs following optogenetic inhibition of MD-ACC pathway. Representative data from Cell #38(B2) about shock responding activity during optogenetic inhibition of MD-ACC pathway. Top panel) Raster plots of calcium transients before and after the shock moment over 10 shocks during red light-OFF and light-ON condition. Black line plots indicate the event timing of calcium transients. Yellow color bar indicates the shock period (2 s). Red color bars indicate the red-LED ON period. Bottom panels) Frequency distribution of mean spike rate determined by 1000 times shuffle. Dashed line indicates 95th percentile of the frequency distribution. Blue line indicates mean spike rate observed during shock moment during red light-OFF and light-ON condition. Total 12 cells were identified as SRCs during light-ON, but not light-OFF, condition.

## Data Availability

Datasets used in this study and source data are available at https://github.com/indra161/MD-ACC_OF-Study_Nair-Ramesh. Custom-made MATLAB scripts used in the analysis of current data is available at https://github.com/indra161/MD-ACC_OF-Study_Nair-Ramesh for reference.

## Abbreviations

OF: Observational fear
ACC: Anterior cingulate cortex
MD: Mediodorsal thalamus
BLA: Basolateral amygdala
AM: Anteromedial thalamus
CTB: Cholera toxin B
IACUC: Institutional animal care and use committee
GPIO: General purpose input/output
ROI: Region of interest
IDPS: Inscopix data processing software
SEM: Standard error of the mean
