# Supplementary material for: Roles of mediodorsal thalamus in observational fear-related neural activity in mouse anterior cingulate cortex
Source: Mol Brain. 2025 Feb 25;18:14. doi: 10.1186/s13041-025-01188-9 (PMC11853286; doi:10.1186/s13041-025-01188-9)
Supplement: Supplementary file 1 — Supplementary Material 1: Supplemental fig. 1: (related to Fig. 2). Trial by trial visualization of calcium activity and calcium transients in SRCs and Non-SRCs. a) Representative data of SRCs from Cell #12 (B2) and Cell #58 (B2). Top panel: mean calcium transient over all 10 demonstrator shock moments in red light-OFF condition. Bottom panels (Trial 1–10): calcium transients during each demonstrator shock period. Significant spikes marked with blue stem plot. Yellow color bar indicates the shock period (2 s). b) Representative data of Non-SRCs from Cell #13 (B2) and Cell #29 (B2). Top panel: mean calcium transient over all 10 demonstrator shock moments in red light-OFF condition. Bottom panels (Trial 1–10): calcium transients during each demonstrator shock period. Significant spikes marked with blue stem plot. Yellow color bar indicates the shock period (2 s). Supplemental fig. 2: (related to Fig. 3). Activation of ACC SRCs following optogenetic inhibition of MD-ACC pathway. Representative data from Cell #38(B2) about shock responding activity during optogenetic inhibition of MD-ACC pathway. Top panel) Raster plots of calcium transients before and after the shock moment over 10 shocks during red light-OFF and light-ON condition. Black line plots indicate the event timing of calcium transients. Yellow color bar indicates the shock period (2 s). Red color bars indicate the red-LED ON period. Bottom panels) Frequency distribution of mean spike rate determined by 1000 times shuffle. Dashed line indicates 95th percentile of the frequency distribution. Blue line indicates mean spike rate observed during shock moment during red light-OFF and light-ON condition. Total 12 cells were identified as SRCs during light-ON, but not light-OFF, condition. [file 13041_2025_1188_MOESM1_ESM.docx]

**
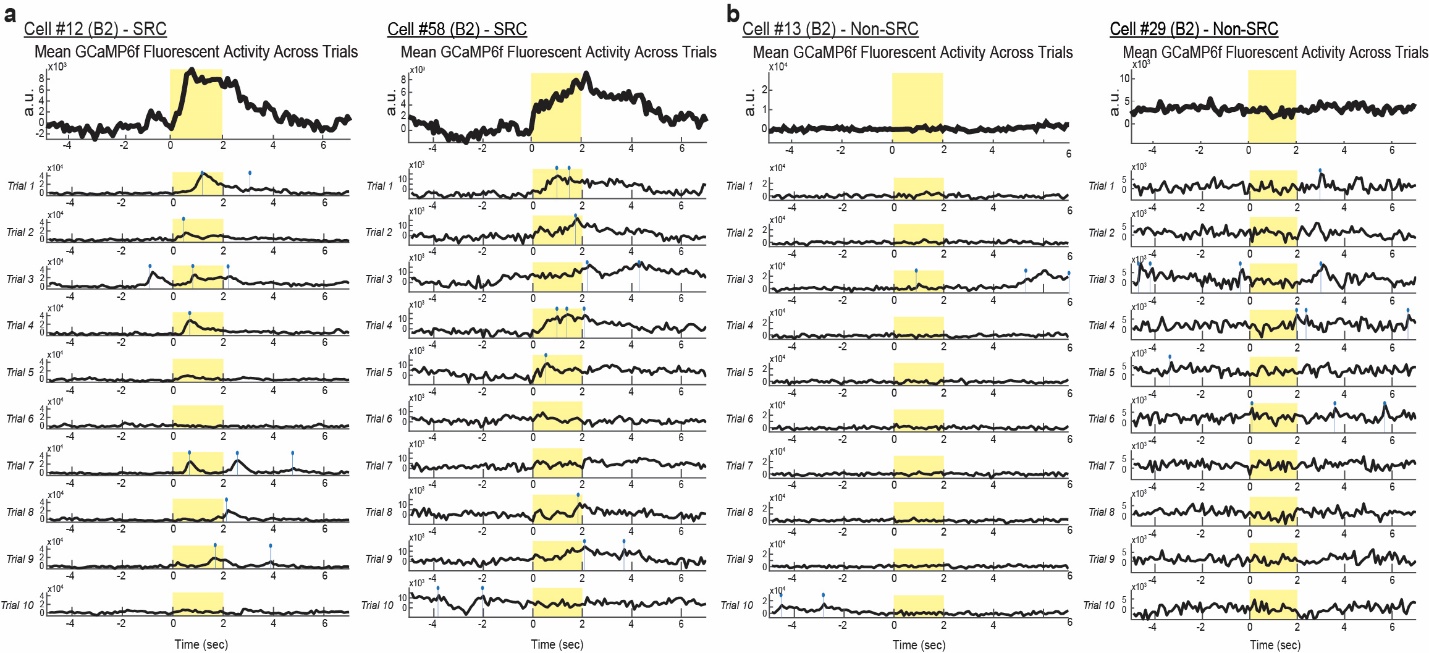
**

**Supplemental Figure 1 (related to Figure 2). Trial by trial visualization of calcium activity and calcium transients in SRCs and Non-SRCs. a)** Representative data of SRCs from Cell #12 (B2) and Cell #58 (B2). Top panel: mean calcium transient over all 10 demonstrator shock moments in red light-OFF condition. Bottom panels (Trial 1-10): calcium transients during each demonstrator shock period. Significant spikes marked with blue stem plot. Yellow color bar indicates the shock period (2 second). **b)** Representative data of Non-SRCs from Cell #13 (B2) and Cell #29 (B2). Top panel: mean calcium transient over all 10 demonstrator shock moments in red light-OFF condition. Bottom panels (Trial 1-10): calcium transients during each demonstrator shock period. Significant spikes marked with blue stem plot. Yellow color bar indicates the shock period (2 second).

**
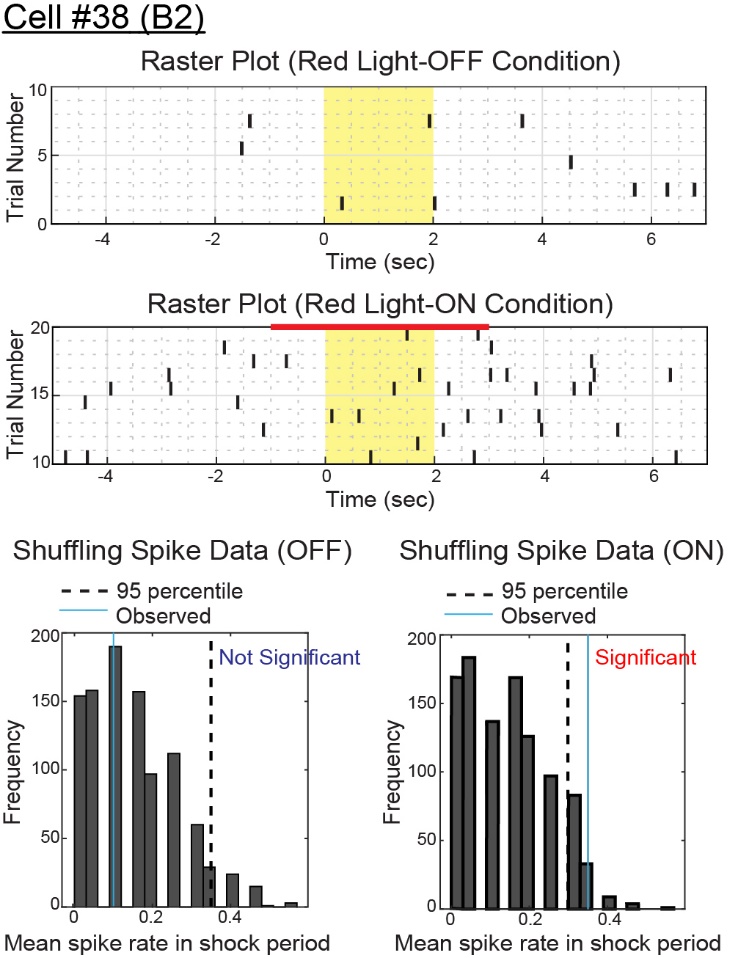
**

**Supplemental Figure 2 (related to Figure 3). Activation of ACC SRCs following optogenetic inhibition of MD-ACC pathway.** Representative data from Cell #38(B2) about shock responding activity during optogenetic inhibition of MD-ACC pathway. Top panel) Raster plots of calcium transients before and after the shock moment over 10 shocks during red light-OFF and light-ON condition. Black line plots indicate the event timing of calcium transients. Yellow color bar indicates the shock period (2 second). Red color bars indicate the red-LED ON period. Bottom panels) Frequency distribution of mean spike rate determined by 1000 times shuffle. Dashed line indicates 95^th^ percentile of the frequency distribution. Blue line indicates mean spike rate observed during shock moment during red light-OFF and light-ON condition. Total 12 cells were identified as SRCs during light-ON, but not light-OFF, condition.
